# Data-driven audiogram classifier using data normalization and multi-stage feature selection

**DOI:** 10.1038/s41598-022-25411-y

**Published:** 2023-02-01

**Authors:** Abeer Elkhouly, Allan Melvin Andrew, Hasliza A Rahim, Nidhal Abdulaziz, Mohd Fareq Abd Malek, Shafiquzzaman Siddique

**Affiliations:** 1 Faculty of Electronic Engineering & Technology, Universiti Malaysia Perlis, 02600 Arau, Perlis Malaysia; 2 Advanced Communication Engineering, Centre of Excellence (ACE), Universiti Malaysia Perlis, 01000 Kangar, Perlis Malaysia; 3 Faculty of Engineering and Information Sciences, the University of Wollongong in Dubai, 20183 Dubai, United Arab Emirates; 4 Faculty of Electrical Engineering & Technology, Universiti Malaysia Perlis, 02600 Arau, Perlis Malaysia; 5 School of Engineering and Physical Sciences, Heriot Watt University, Dubai Knowledge Park, 38103 Dubai, United Arab Emirates; 6 Biotechnology Research Institute, Universiti Malaysia Sabah, 88400 Kota Kinabalu, Sabah Malaysia

**Keywords:** Health care, Medical research, Engineering

## Abstract

Audiograms are used to show the hearing capability of a person at different frequencies. The filter bank in a hearing aid is designed to match the shape of patients’ audiograms. Configuring the hearing aid is done by modifying the designed filters’ gains to match the patient’s audiogram. There are few problems faced in achieving this objective successfully. There is a shortage in the number of audiologists; the filter bank hearing aid designs are complex; and, the hearing aid fitting process is tiring. In this work, a machine learning solution is introduced to classify the audiograms according to the shapes based on unsupervised spectral clustering. The features used to build the ML model are peculiar and describe the audiograms better. Different normalization methods are applied and studied statistically to improve the training data set. The proposed Machine Learning (ML) algorithm outperformed the current existing models, where, the accuracy, precision, recall, specificity, and F-score values are higher. The reason for the better performance is the use of multi-stage feature selection to describe the audiograms precisely. This work introduces a novel ML technique to classify audiograms according to the shape, which, can be integrated to the future and existing studies to change the existing practices in classifying audiograms.

## Introduction

The World Health Organization (WHO) estimates that by 2050, nearly 2.5 billion people are projected to have some degree of hearing loss, which, poses an annual global cost of USD 980 billion^1^. Daniela Bagozzi, a WHO Senior Information Officer, wrote an article to make a call for the private sector to provide affordable hearing aids in developing countries (as their current cost ranges from USD 200 to over USD 500)^2^. In addition, the Healthline Organization reported that a set of hearing aids might cost USD 5000^3^. The impact of hearing loss on nations’ economies is estimated USD 750^1^. The process of fitting hearing aids is tiring and consuming in time as it depends on many trials which require the patient to be highly responsive. A study stated that only 50–60% are satisfied with their hearing aids use^4^. The significant increase in the numbers of individuals with hearing loss, the high cost of hearing aids, the burden of hearing loss on the global economy, low customer satisfaction with their hearing aids and the severe shortage of numbers of audiologists who are very rare and hard to find in rural areas^5,6^, motivated the authors to come up with a technical solution for all the mentioned problems. The main idea here is to use the Machine Learning (ML) to facilitate the whole process for the patients and the hearing aid designers.

**Figure 1:**
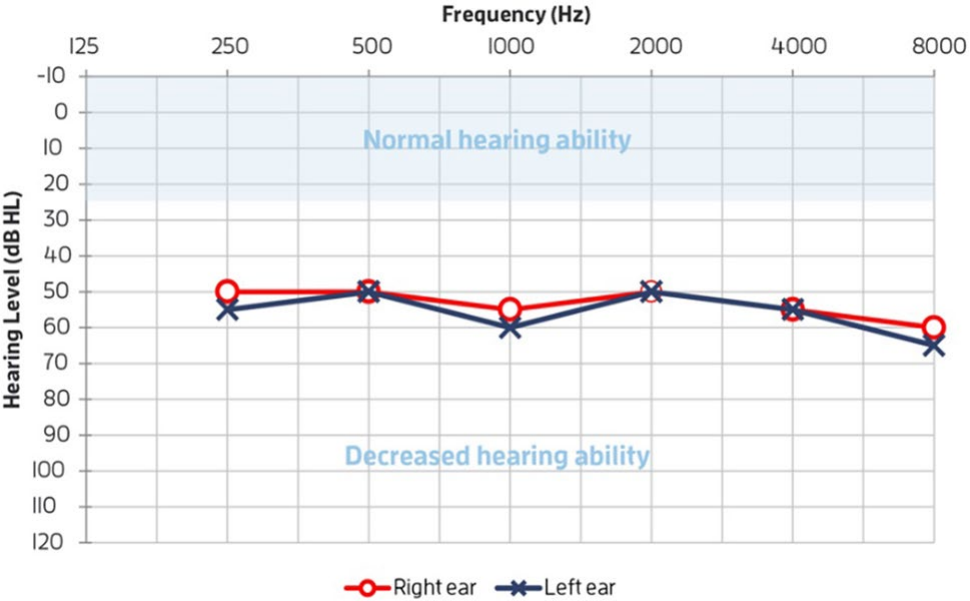
Audiogram to show the hearing ability of left and right ears. X left ear, O right ear.

The audiogram is used to display the hearing test results and it shows the hearing capability of a person at different frequencies that range from 250 to 8000 Hz. Figure 1 shows the audiogram that is obtained from an audiometer, showing the hearing levels are in dB at different frequencies in Hz. The hearing ability of both the left and the right ears is measured, X is to indicate the left ear while O marks for the right ear^7^.

To the best of our knowledge, the studies to classify audiograms are few and far apart, especially, the ones that applies intelligent solutions. In 2016, Rahne et al., have built an excel sheet as an audiogram classifier with the pre-set inputs, that can be defined according to inclusion criteria in the clinical trial. This tool provides inclusion decision for audiograms based on the predefined audiological criteria^4^. Then, in 2018, Sanchez et al. have classified the hearing tests data in two stages. The first stage is the unsupervised learning to define trends and spot patterns in data obtained from different hearing tests. In the second stage, a supervised learning algorithm is built based on different hearing tests to classify hearing loss into 4 types related to sensitivity and clarity loss. It used different types of hearing tests, not only audiograms, to classify different collected data to detect the type of hearing loss that was not captured by the audiogram^4^.

Belitz et al., in 2019 have also combined the unsupervised and the supervised machine learning methods to map audiograms into a small number of hearing aid configurations. The target of this study was to use these configurations as a starting point for the hearing aid fitting. This method was applied in two steps, the first one started by performing different unsupervised clustering algorithms to determine a limited number of pre-set configurations for a hearing aid. The centroids of the clusters were chosen to represent fittings targets, which can be used as the starting configurations for the hearing aid adjustments for each individual. The second step was to assign each audiogram to a class based on the results from the first stage of comfort target clustering. Various supervised machine learning techniques were used to assign each audiogram to a pre-set configuration. The classifier accuracy of the second stage was low when they selected a single configuration and it was improved when they allowed two configurations for each audiogram^8^.

In 2018, Charih et al. developed a machine learning classifier using the unsupervised learning to cluster the audiograms^5^. In this work, audiograms were clustered with the target to make them maximally informative audiograms. Then, the clustered data was prepared to be a good training set for supervised machine learning classifiers. They built an approach to get a set of non-redundant unannotated audiograms with minimal loss of information from a very big data set. In 2020, the same group used the data preparation procedure carried out by them to produce a machine learning classifier. They applied supervised ML to 270 audiograms annotated by three experts in the field. The results have good accuracy to annotate the audiograms concisely in terms of shape, severity and symmetry^5^. The classifier can be integrated as a mobile application to help the user to describe the audiogram concisely, so that, it can be interpreted by non-experts. The classifier outputs can be used by non-experts to decide if the patient needs to be checked by a specialist. It can resolve partially the problem of having a shortage of specialists and it can be the first step towards a more sophisticated algorithm to help experts in the field of audiology.

Crowson et al., used deep learning convolutional neural network architecture to classify audiograms of normal hearing, sensorineural, conductive, and mixed hearing loss. The audiograms were converted to jpeg formatted picture files. Image transformation techniques were used to increase the number of images available as training data for the classifier. Image rotation, wrapping, contrast, lighting and zoom were applied to the audiogram images in the training set. They achieved 97.5% accuracy of their model to classify hearing loss types based on features extraction of the audiograms^6^. In the research, the study aimed at classifying audiograms to detect the cause of hearing loss, which is not helpful in configuring hearing aids. In addition, the visual format of the audiograms is not a level-based representation, which, makes the shape of audiograms differs depending on the scales used to represent the frequencies and the threshold levels^6^.

Musiba^9^ has classified audiograms based on UKHSE (United Kingdom Health and Safety Executive) categorization scheme. The sum of pure tone audiometry test hearing levels at frequencies 1 kHz, 2 kHz, 3 kHz, 4 kHz and 6 kHz, were obtained. Then, compared with the figures set by UKHSE and classified as one of the following: acceptable hearing ability, mild hearing impairment, poor hearing, or rapid hearing loss. The aim of this classification was to prompt proper actions to prevent noise-induced hearing loss. The annotation process was carried out by experts in the field, applying the UKHSE standards. Cruickshanks and his team^10^ made a longitudinal study on how the shape of audiograms changes over time. The follow up was carried out based on four stages; 1993–1995, 1998–2000, 2003–2005, and 2009–2010. The audiograms were classified into eight levels and the change in hearing ability over time was recorded based on these classes. Musiba^9^ and Cruickshanks^10^ did not implement any intelligent solutions as they counted on the experience of the specialists in the field.

Another technique that was used in many applications to improve the data quality and to enhance the accuracy of the produced ML model, is the use of multi-stage feature selection classifiers. This technique has been used in many applications to enhance the accuracy of the classifiers^11^. Cerrada proposed a multi-stage feature selection mechanism using genetic algorithms. It was proposed in each stage a new subset of the best features related to the classifier performance in a supervised environment. The selected features are optimized at each stage and used as input for a neural network classifier in the next step^12^. Andrew optimized the classification accuracy for the early fire detection algorithm by multi-stage feature selection. These stages are; normalized feature extraction, feature selection based on best classification accuracy, data dimension reduction then feature fusion^11^. Vijayasarveswari used multi-stage feature selection in early breast cancer size prediction. The algorithm has four stages; data normalization, feature extraction, data dimensional reduction, and feature fusion^13^.

This paper is organized as follows. Firstly, in “Introduction”, the method used in this research is discussed, where, different stages implemented are explained in details. The findings from the authors’ previous work in this research are discussed as it explains how the data set used in this paper is annotated. The data pre-processing, and the ML model feature extraction are elaborately discussed in “Methods”. This is followed by the results section, where, the model is optimized and the model performances are evaluated. Finally, a discussion of the results, conclusion, and prospects for future work are presented in “Discussion and conclusion”.

## Methods

### Method description

The first stage of this study was done to cluster large data set of 28,244 audiograms using vector quantization of size 60^14^. The authors given permission to use the data from Bisgaard et al. for this study. The data in the paper were prepared based on International Electrotechnical Commission (IEC) standard 60118-15, and the measurement method was approved by local ethical committee. The data availability statement is provided under subsection ‘Data availability’.

Then, ML unsupervised spectral clustering was applied to classify these audiograms into classes according to similarity in the shape^15^. The authors clustered the quantized data into 7–11 clusters, and based on the statistical test conducted, 10 clusters are selected as the optimum number of clusters as this number of clusters gives the highest criteria values. The data’s nature is of high overlapping which means the different shapes cannot be captured by a low number of classes. A detailed description to the authors’ previous work on unsupervised spectral clustering algorithm, how it was implemented and its performance evaluation can be found in^15^.

**Figure 2:**
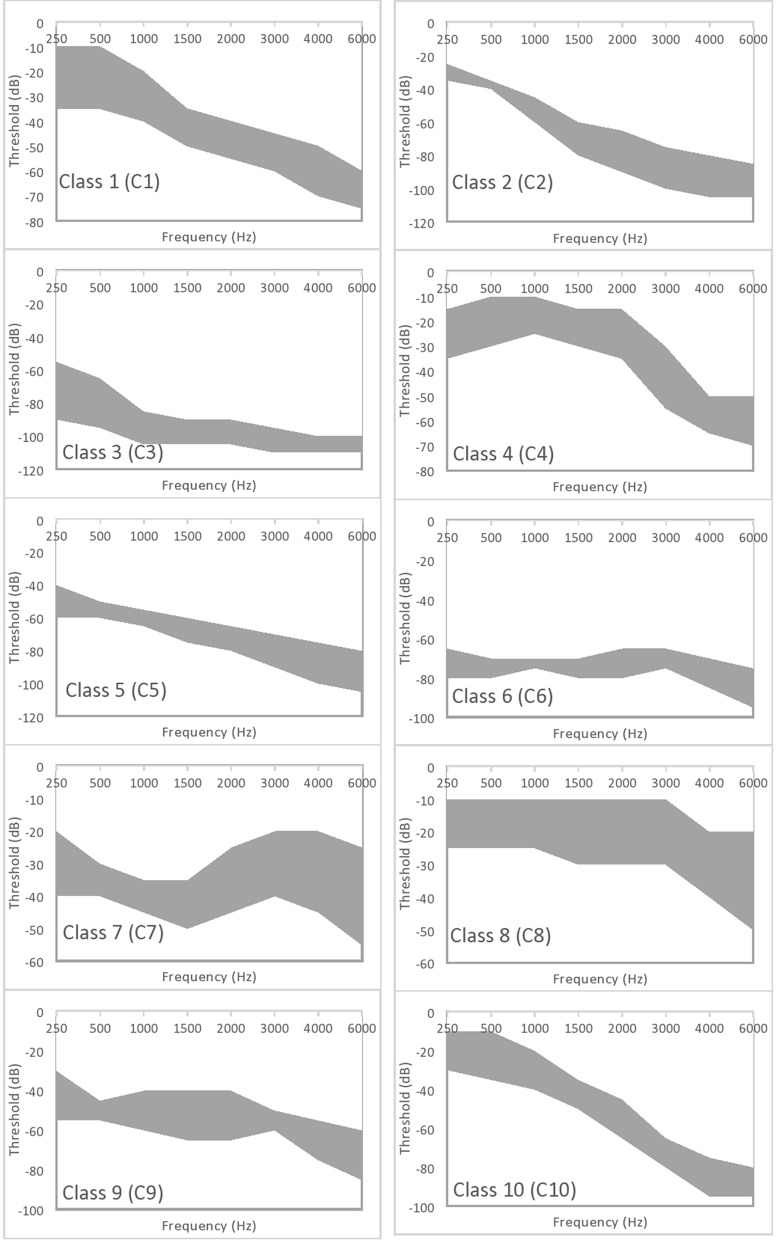
Audiograms clusters based on spectral clustering^15^.

The detected shapes by this unsupervised clustering are shown in Fig. 2. These clusters cannot be confined to a discrete level to represent each. But, as can be seen in the figures, the information of a shade with a certain shape can be useful in the analysis. If necessary to represent each cluster by a single audiogram, the median values of the shaded area or using polynomial regression as done in the author’s previous work^16^ can be used. Different hearing levels ranges are used to display different clusters, where, a maximum value of − 60 dB is used for classes C7 and C8, − 80 dB for C1 and C4, − 100 dB for C6, C9 and C10 and − 120 dB for C2, C3 and C5. Unifying the maximum value will not affect the shapes of the clusters as linear scaling is used to display the data. The authors chose to use different ranges to clearly show the difference in the detected shapes.

**Figure 3:**
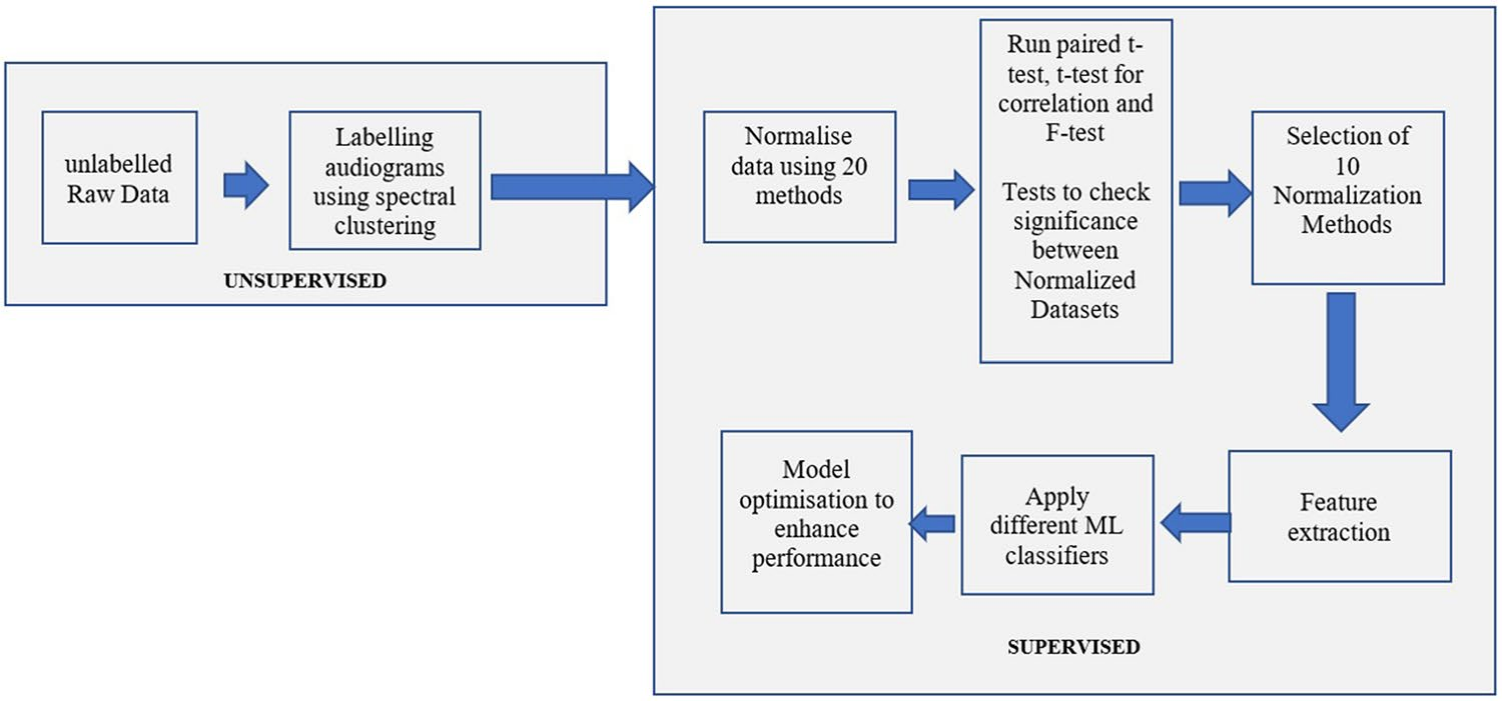
Stages to build the ML model.

In the current study, a supervised machine learning technique is developed based on the annotated data from the unsupervised spectral clustering done by^15^. This data source contains samples of clean data with no outliers, no redundant values, no missed values and it is in the format that is suitable for use. The hearing levels were measured at the frequencies 250 Hz, 500 Hz, 1 kHz, 1.5 kHz, 2 kHz, 3 kHz, 4 kHz, and 6 kHz. Even though the number of clustered audiograms is small to train a credible ML model, the problem is overcome by performing different normalization techniques to the data. These normalized sets of data are inspected statistically to select the ones without redundancy and without out of shape due to the normalization to train the ML model. The research work flow is described in Fig. 3.

### Data normalization

Data pre-processing in this research starts with data normalization^17^, where, 20 different techniques are applied to the data^18^. This step is important to create a model with good accuracy^19^. In this study, 20 normalization techniques are applied to the data which are; Z-Score Normalization, Linear Scaling, Binary Normalization, Bipolar Normalization, Min-Max Scaling, t-Score Normalization, Differential Moment Normalization, Variation Normalization, Decimal Inverse Logarithmic Scaled Normalization, Absolute Percentage Error Normalization (APE) formula 1, Absolute Percentage Error Normalization (APE) formula 2, Arctan APE formula 1, Arctan APE formula 2, Gaussian Normalization, Relative Sum Squared Value (RSSV), Relative Logarithmic Sum Squared Value (RLSSV), Relative Mean Normalization, Relative Standard Deviation Normalization, Relative Interquartile Normalization, and Robust Normalization. These normalization formulas are defined as in Table 1 and are calculated with the aid of MATLAB R2019b and Excel. Detailed information related to the formula is presented the in associated reference given. The quantized data by Bisgaard of size 60, is normalized using the above methods. This process gives 1200 normalized audiograms, which, are studied statistically in the next stage to select the best 10 normalized methods out of 20 normalization methods, which, gives 600 normalized audiograms.

**Table 1:** Normalization methods formulas.

No.	Normalization	Equation	References
1	Z-score normalization	$x' = \frac{x-\mu }{\sigma }$	^18^
2	Linear scaling	$x' = \frac{x-min}{max-min}$	^18^
3	Binary normalization	$x' = (\frac{0.8(x-min)}{max-min})+0.1$	^20^
4	Bipolar normalization	$x' = (\frac{1.8(x-min)}{max-min})-0.9$	^20^
5	Min-Max scaling	$x' = \frac{(x-min)(max_{n}- min_{n})}{max-min} + min_{n}$	^21^
6	t-score normalization	$x' = \frac{x- \mu }{ \frac{\sigma }{\sqrt{n}}}$	^22^
7	Differential moment normalization	$M_{i} = \frac{1}{ N^{2} } ( \sum _{i=1}^N x_{i}) ^{2} - x_{i} ^{2}$	^23^
8	Variation normalization	$C_{V,i} = \frac{ \sigma }{ \mu } x_{i}$	^22^
9	Decimal inverse logarithmic scaled normalization	$x' = 10^{-12} 10^{0.1x} 10^{7}$	^24^
10	Absolute percentage error normalization (APE) formula 1	$x' = (\frac{{\overline{x}}-x_{i}}{\frac{({\overline{x}}+x_{i})}{2}})$	^25^
11	Absolute percentage error normalization (APE) formula 2	$x' = (\frac{{\overline{x}}-x_{i}}{{\overline{x}}} )$	^26^
12	Arctan APE formula 1	$x' = arctan(\frac{{\overline{x}}-x_{i}}{ \frac{{{\overline{x}}+x_{i}}}{2} } )$	^26^
13	Arctan APE formula 2	$x' = arctan(\frac{{\overline{x}}-x_{i}}{ {{\overline{x}} } } )$	^26^
14	Gaussian normalization	$x' = \frac{1}{ \sqrt{2 \Pi \sigma ^{2} } } exp(- \frac{ ( x_{i}- \mu ) ^{2} }{2 \sigma ^{2} })$	^22^
15	Relative sum squared value (RSSV)	$x' = \frac{x}{ \sum (x^{2}) }$	^11^
16	Relative logarithmic sum squared value (RLSSV)	$x' = \frac{log(x)}{ log(\sum x^{2}) }$	^11^
17	Relative mean normalization	$x' = \frac{x}{ {\overline{x}} }$	^23^
18	Relative standard deviation normalization	$x' = \frac{x}{ \sigma }$	^23^
19	Relative interquartile normalization	$x' = \frac{x}{ IQR }$	^23^
20	Robust normalization	$x' = \frac{(x-median)}{ IQR }$	^18^

### Normalized data set selection

**Figure 4:**
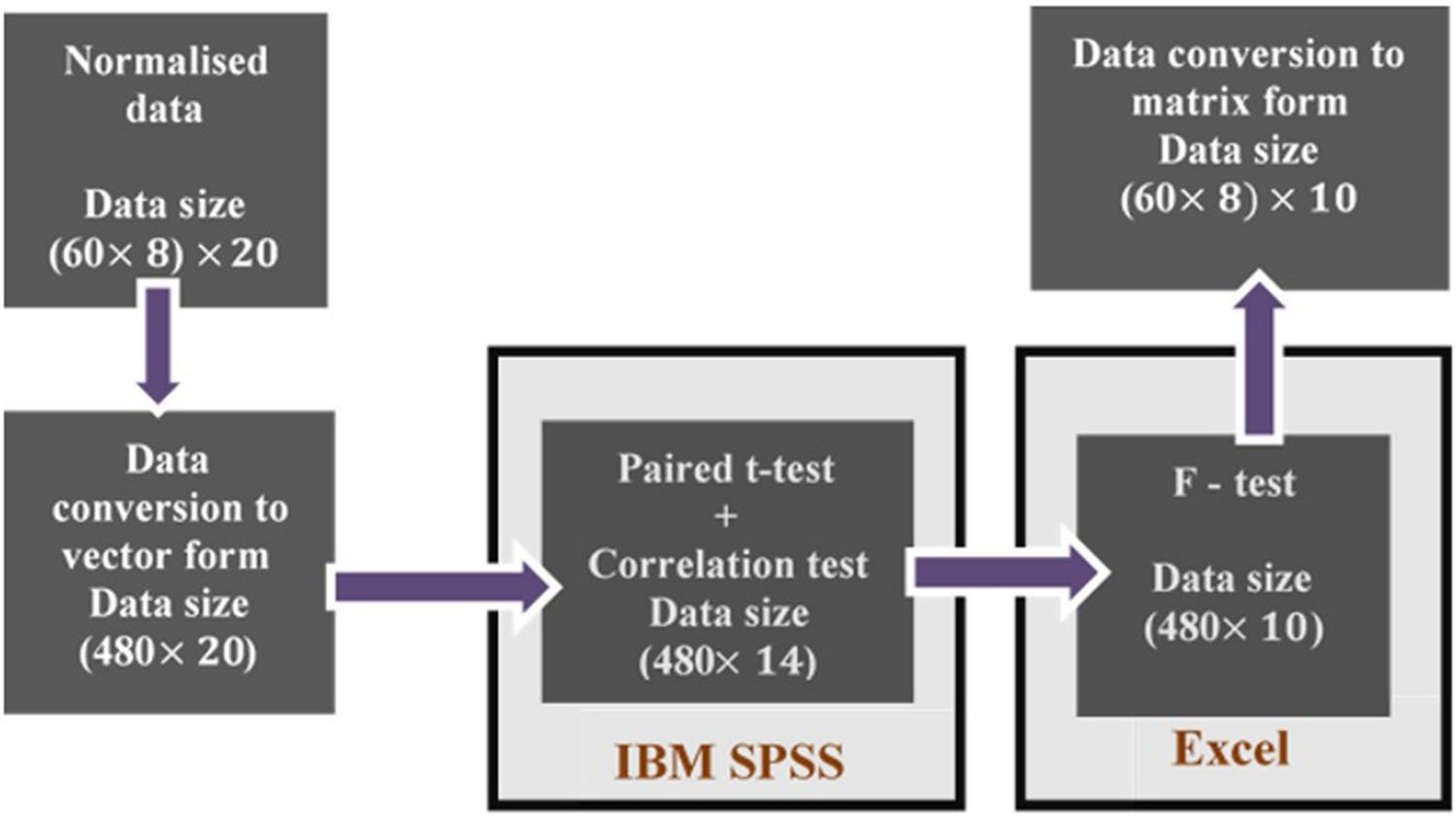
Normalization methods selection steps.

Different statistical analyses are applied to the 20 sets of normalized data. The hypotheses of these analyses are built to infer if normalized methods lead to data redundancy or inconsistency in data pattern. The aim of this process is to keep the normalized data sets which produce data without redundancy. In addition, these 10 normalized data sets must not lead to any change in the relative levels of the audiograms to maintain their shapes. This process passes through 3 different statistical analyses; paired t-test for mean difference, t-test for correlation significance, and then, F-test for data variability as shown in Fig. 4. These tests are applied to the normalized 60 audiograms, resulting from Bisgaard quantized data.

These analyses were run using IBM SPSS Statistics 23. Then, the data normalization methods are compared in pairs using paired t-test at 0.05 level of significance, to test the hypothesis:

*H0: there is no difference in the mean value of data normalized with technique A and data normalized with technique B*.

*H1: there is a difference in the mean value of data normalized with technique A and data normalized with technique B*.

The t-test statistic value is calculated using Eq. (1), which, gives the p-value. Then, a decision should be made according the decision rule: if p-value $< \alpha$, reject H0. This should be repeated for two different normalization methods at a time which means, this will be done $C_2^{20}=190$ times.

1 $$\begin{aligned} t_{STAT} = \frac{{\overline{D}}-\mu _{D}}{\frac{ S_{D}}{\sqrt{m}}} \end{aligned}$$

where $\mu _D$ is the hypothesized mean difference, which, in this case 0 and m = 60 $\times$ 8. Assume $x_{11},x_{12},\ldots ,x_{1m}$ are the data values normalized with technique A and $x_{21},x_{22},\ldots ,x_{2m}$ belong to data set normalized with technique B, then $S_{D}$ can be found using Eqs. (2), (3) and (4):

2 $$\begin{aligned} D_{i}= & {} x_{1i}-x_{2i} \end{aligned}$$

3 $$\begin{aligned} {\overline{D}}= & {} \frac{ \sum _{i=1}^m D_{i} }{m} \end{aligned}$$

4 $$\begin{aligned} S_{D}= & {} \sqrt{ \frac{ \sum _{i=1}^m ( D_{i}- {\overline{D}})^{2}}{m-1}} \end{aligned}$$

Applying the paired t-test, 6 normalization data sets are found to be redundant. As a second step, the output from the paired t-test will go through another t-test for correlation, to test if the produced normalized data sets are significantly correlated at 0.05 level of significance. These hypotheses are:

*H0:* $\rho$=0; *there is no significant correlation between data normalized with technique A and data normalized with technique B*

*H1:* $\rho \ne$0; *there is significant correlation between data normalized with technique A and data normalized with technique B*

The t-test statistic for correlation is calculated with Eq. (5) and the decision to reject H0, if p value $> \alpha$.

5 $$\begin{aligned} t_{STAT} = \frac{r- \rho }{ \sqrt{ \frac{1- r^{2} }{m-2} } } \end{aligned}$$

where $$r= \frac{cov(X,Y)}{ S_{X} S_{Y} }$$; $$cov(X,Y)= \frac{ \sum _{i=1}^m ( X_{i}- {\overline{Y}})(X_{i}- {\overline{Y}}) }{m-1}$$;$$S_{X} = \sqrt{ \frac{ \sum _{i=1}^m ( X_{i}- {\overline{X}}) ^{2} }{m-1} }$$;$$S_{Y} = \sqrt{ \frac{ \sum _{i=1}^m ( Y_{i}- {\overline{Y}}) ^{2} }{m-1} }$$.

$X_i$ belongs to data normalized with technique A & ${\overline{X}}$ is the mean value of $X_i$ values and $Y_i$ belongs data normalized with technique B and the corresponding mean is ${\overline{Y}}$ and m = 60.

The third test is to check the data variability with F-test with the following hypotheses:

*H0: the two data sets have equal variances.*

*H1: the two data sets with variances not equal.*

The F-test statistic is calculated with Eq. (6) and the decision to reject H0, if $F_{STAT} >F_{(\alpha /2)}$,

6 $$\begin{aligned} F_{STAT} = \frac{S_1^2}{S_2^2} \end{aligned}$$

where $S_1^2$ = variance of sample 1 with larger sample variance, and $S_2^2$ = variance of sample 2 with smaller sample variance.

**Figure 5:**
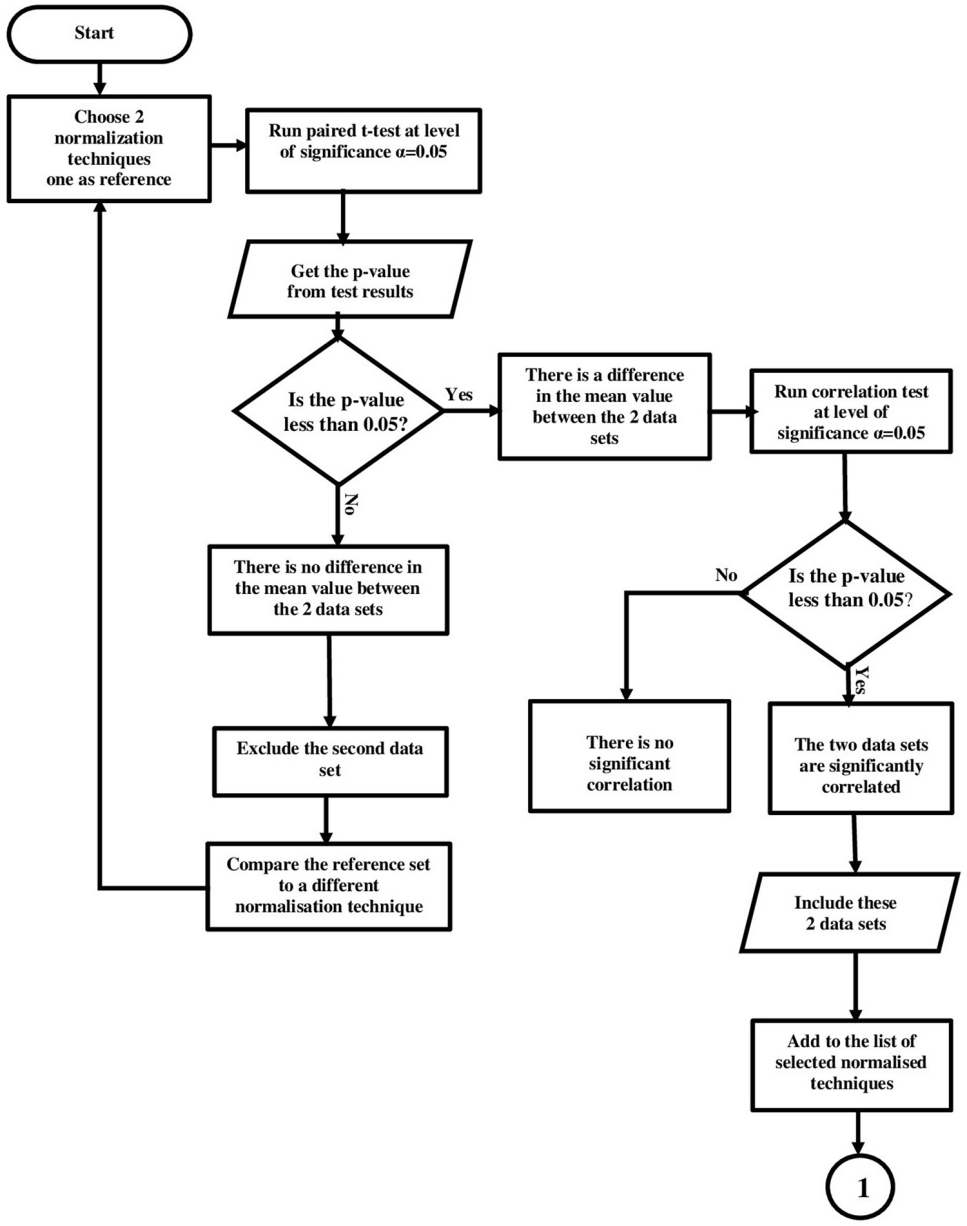
Statistical analysis sequence flow chart (Part 1).

**Figure 6:**
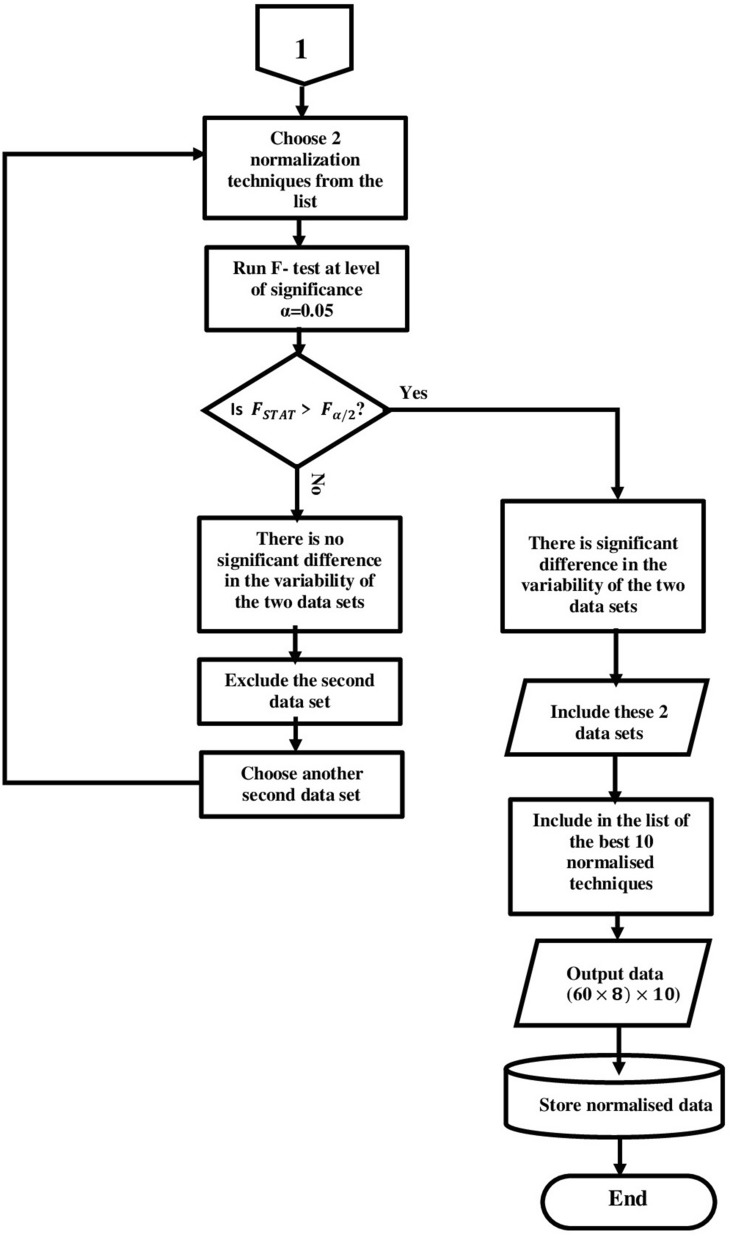
Statistical analysis sequence flow chart (Part 2).

The sequence of the statistical analysis is illustrated with the flowchart in Figs. 5 and 6.

### Feature extraction using modeling

This step is very important as it is the key of having a good ML model with high prediction accuracy. Predictors are the best guess features that are needed to be extracted from the data such that the main patterns in data are detected by the ML classifier. The derived features of audiograms are calculated based on the hearing levels at frequencies from 250 Hz to 6 kHz. The features are the average of hearing levels at all frequencies (A1), the average of hearing levels at frequencies 0.5 kHz, 1 kHz, 1.5 kHz and 2 kHz (A2), the average hearing levels at the frequencies 3 kHz, 4 kHz and 6 kHz. The steepness of the audiograms for the steep sloping group can be detected by SS1, SS2, and SS3 as described in Eqs. (10)–12. The other statistical values that considered to describe each audiogram are; minimum, maximum, difference between these two values, the standard deviation and the median. Another set of features are extracted by calculating the correlation coefficient between each audiogram and the ten standard levels used by Bisgaard^14^, refer to Eq. (5). Then, each audiogram is described by polynomials of degrees 3, 4 and 5 and the residual is calculated using MATLAB ‘polyfit’ function. Coefficients of the polynomial of degree 5 are considered as the least square residuals found to be the lower among the three cases. Hence, six features are added; the 5 coefficients of different exponents and the constant term. The calculated residual is an added feature, where, the sum of the squares of the differences between the original audiogram and the polynomial that fits the audiogram is calculated. The steepness is also added as a feature. Value 1 is assigned if the difference between the maximum and the minimum exceeded 60 dB, and, otherwise, 0 is assigned. Assume hearing levels are denoted by $H_1, H_2, H_3, H_4, H_5, H_6, H_7$, and $H_8$. The average levels and variations are calculated using Eqs. (7)–(14).

7 $$\begin{aligned} A1= & {} \frac{ \sum _{i=1}^8 H_{i} }{8} \end{aligned}$$

8 $$\begin{aligned} A2= & {} \frac{ \sum _{i=2}^5 H_{i} }{4} \end{aligned}$$

9 $$\begin{aligned} A3= & {} \frac{ \sum _{i=6}^8 H_{i} }{3} \end{aligned}$$

10 $$\begin{aligned} SS1= & {} \frac{ H_{4}- H_{8} }{4.5} \end{aligned}$$

11 $$\begin{aligned} SS2= & {} \frac{ H_{3}- H_{6} }{2} \end{aligned}$$

12 $$\begin{aligned} SS3= & {} \frac{ H_{2}- H_{5} }{1.5} \end{aligned}$$

13 $$\begin{aligned} S_{N1}^2= & {} \frac{ \sum _{i=1}^m ( H_{i}- H_{iN1})^2 }{7} \end{aligned}$$

14 $$\begin{aligned} S_{N2}^2= & {} \frac{ \sum _{i=1}^m ( H_{i}- H_{iN2})^2 }{7} \end{aligned}$$

where $S_{N1}^2$ (variation with respect to N1), and $S_{N2}^2$ (variation with respect to N2). Similarly, it can be calculated for audiogram configurations N3–N7 and with respect to steep- sloping groups S1–S3. Hence, 39 features will be used; three average hearing levels A1–A3, three slopes to measure steepness SS1–SS3, five statistical measures, ten correlation coefficients, six polynomial coefficients, residual, the steepness, and 10 variances ($S_{N1}^2$ to $S_{S3}^2$) to measure the variation with respect to the standard levels.

## Results

### Selection of normalization method based on statistical analysis

Different normalization methods are compared in pairs with a paired t-test to check if there is a significant difference in the mean value of the normalized audiograms at $\alpha$ = 0.05. Variability test is the first step to remove the redundant normalized methods. At this stage, 6 normalized methods are removed as the p value >$\alpha$, the Z-score was chosen as the reference set to start running the tests using SPSS. These normalization methods are removed statistically; Bipolar Normalization, Arctan APE formula 1, Arctan APE formula 2, Gaussian Normalization, Absolute Percentage Error Normalization (APE) formula 2, and Relative Sum Squared Value (RSSV). Then, the data variability is checked with the F-test. It is found that four normalization methods indicated that the audiograms variability is the same, and thus, are removed. The additional excluded ones are; t-Score Normalization, Differential Moment Normalization, Variation Normalization, and Decimal Inverse Logarithmic Scaled Normalization.

Finally, the correlation test is conducted to check whether the correlation between the normalized audiograms is significant at $\alpha$ = 0.05 or not, which, indicated that the selected ten normalization methods are significantly correlated. This result shows that there are no changes in the relative levels of the audiograms and as a consequence, their shapes are maintained using the selected normalization methods. The authors considered the following normalization methods show different means and variability but correlated: Z-Score Normalization, Linear Scaling Normalization, Binary Normalization, Min-Max Normalization, Absolute Percentage Error (APE) Normalization, Relative Logarithmic Sum Squared Value (RLSSV), Relative Mean, Relative Standard Deviation, Relative Interquartile, and Robust Normalization.

### Assigning new classes using spectral clustering

The authors as discussed in the previous paper^15^ decided to remove the set of audiograms that represent normal hearing levels. These levels are removed since the algorithm is developed to assist in configuring the hearing aids for patients who are experiencing hearing loss. The selected 55 audiograms of Bisgaard quantized data are clustered into 10 classes using spectral analysis. The spectral clustering algorithm^27^ is a graph-based technique to find k clusters in data^28^. In^15^, the authors grouped the audiograms according to the similarity in the ten clusters’ shape. These audiograms were furtherly cleaned by removing the audiograms that were wrongly assigned or weakly clustered to the clusters. This data cleaning process led to a better clusters’ evaluation criterion as presented in this research. 49 audiograms were clustered according to the similarity in shape with high indices of different evaluation criteria such as; Silhouette (SI), Calinski–Harabasz (CH), and Davies–Bouldin (DB) criteria values. The authors believe that the existing audiogram classes used in audiology are not suitable for providing the clusters that can be technically used as a reference by the specialists in the field, such as the audiologists, the hearing aid specialists, and the hearing aid designers.

### Classification using supervised machine learning

The process starts by preparing the training data to be used in different classifiers. Firstly, the derived features are calculated using Microsoft Excel and MATLAB R2019b. Microsoft Excel is used to calculate all the features except the polynomial coefficients and the residual. The data classes are labelled by the spectral clustering into 10 classes. The cleaned data (49 samples) are normalized using 10 selected normalization methods, yielding to 490 normalized samples. The original data and the negative thresholds representation of the hearing levels are added to make the data size 588 samples. The model is trained with various classifiers using the Classification Learner Application in MATLAB. The extracted features are studied thoroughly to select the most relevant features that can provide the highest predictive accuracy for the ML model. Firstly, Principal Component Analysis (PCA) is applied but the analysis did not lead to a good model with features reduction. This could be because of the labels not correlated correctly to the features variance. Then, the correlation matrix is created to check the relationship between the variables and, the variables and the labels. This part aims to remove any redundant features or the weakly correlated ones to the labels. This led to the removal of the 10 variances ($S_{N1}^2$ to $S_{S3}^2$). The remaining 29 features are tried iteratively and systematically to remove features that are found to have a minor impact on the model accuracy. The removed features are the steepness measure SS1, and the coefficients of the polynomial of the cubic, quadratic, linear, and constant terms.

By using the trial and error to further reducing the number of features, resulting in the removal of N2, N4, N7, and S1. This results in a reduced number of features to 20, but the maximum model accuracy as low as 85%. To improve the accuracy of the model, different features are mapped to the classes using second-degree and linear functions. The accuracy is checked each time, and the accuracy is improved when a second-degree function is used to map the residual to the classes.

To further improve the model, more complex features were introduced, where, logic operators are applied on the minimum and maximum of A1, A2, A3, medial, minimum–maximum and standard deviation, to produce 10 nested features. These features are described in Table 2. The newly added features significantly improved the model accuracy to 93%. The final total number of feature arrangement is 31, and they are calculated based on the hearing levels of each audiogram.

**Table 2:** The nested features produced to improve the model accuracy.

Feature number	How to classify
22	Minimum and maximum of A1, and A3 of class 1
23	Minimum and maximum of median, and standard deviation of class 2
24	Minimum and maximum of median of class 3
25	Minimum and maximum of A2 and A3 of class 4
26	Minimum and maximum of Median, and standard deviation of class 5
27	Minimum and maximum of Median, and standard deviation of class 6
28	Minimum and maximum of Min-max, and median, of class 7
29	Minimum and maximum of A1 of class 8
30	Minimum and maximum of A3 of class 9
31	Minimum and maximum of A3 of class 10

The model is trained using 5-fold cross-validation with different classifier learners, and, the highest accuracy is resulted for the Fine k-Nearest Neighbor classification algorithm (Fine kNN learner). The performance of the classifier is evaluated by calculating the classification accuracy, precision, recall, specificity, and the F-score ($\beta$ = 1) values. The different normalized datasets are trained individually and for each data set, the five ML performance evaluators are calculated. Performance evaluation results are shown in Table 3 for each normalized data set. The overall performance of the algorithm is calculated based on the average taken for each class from the different normalized data sets. The algorithm performance evaluation is then calculated by getting the averages of the different classes parameters. The performance of every class and the overall classification performance are shown in Table 4. The performance of the proposed algorithm is compared with the performance of the DDAE algorithm^5^. In the research, they used 270 samples of audiograms. The performance of the model is calculated as the average of all the classes.

**Table 3:** Classification performance of each normalized data set.

Data description	No. of audiograms	Accuracy	Precision	Recall	Specificity	F-core $\beta$ = 1	Classifier algorithm
Negative original	49	0.918	0.924	0.925	0.990	0.922	Fine KNN
Positive original	49	0.877	0.887	0.883	0.985	0.875	Fine KNN
Normalized linear scaling	49	0.959	0.980	0.963	0.995	0.968	Fine KNN
Normalized binary	49	0.918	0.956	0.938	0.990	0.943	Fine KNN
Normalized APE 1	49	0.898	0.936	0.9	0.988	0.889	Fine KNN
Normalized RLSSV	49	0.939	0.940	0.938	0.993	0.937	Fine KNN
Normalized MM	49	0.939	0.953	0.950	0.993	0.951	Fine KNN
Normalized mean	49	0.980	0.989	0.980	0.998	0.983	Fine KNN
Normalized std	49	0.898	0.917	0.908	0.988	0.908	Fine KNN
Normalized R IQR	49	0.939	0.950	0.938	0.993	0.940	Fine KNN
Normalized robust	49	0.959	0.975	0.975	0.995	0.975	Fine KNN
Normalized Z-scores	49	0.918	0.951	0.925	0.990	0.929	Fine KNN

**Table 4:** Performance of each class measured as the average of different normalization techniques.

Classes	Proposed algorithm					DDAE			
	Accuracy	Precision	Recall	Specificity	F-score	Accuracy	Precision	Recall	F-score
Class 1	0.896	0.823	0.896	0.959	0.853	0.94	0.8	0.84	0.81
Class 2	1	1	1	1	1	0.81	0.81	0.81	0.81
Class 3	1	1	1	1	1	0.88	0.85	0.82	0.83
Class 4	0.625	0.854	0.625	0.991	0.703	0.96	0.81	0.83	0.81
Class 5	1	1	1	1	1	0.95	0.74	0.62	0.65
Class 6	1	1	1	1	1	0.93	0.83	0.8	0.81
Class 7	0.963	0.901	0.963	0.988	0.925	0.84	0.7	0.71	0.7
Class 8	0.972	0.937	0.972	0.988	0.949	0.84	0.7	0.61	0.62
Class 9	0.896	0.949	0.896	0.989	0.919				
Class 10	1	1	1	1	1				
Performance	0.935	0.946	0.935	0.991	0.935	0.894	0.780	0.755	0.755

All the normalized data sets and the two representations for the audiograms (negative and positive levels) are used as the training data sets. This leads to 49 $\times$ 12 = 588 normalized audiograms applied to the classifier with the same 31 features using Fine kNN learner. The performance of the algorithm is shown in Table 5, and it is compared to the DDAE performance.

**Table 5:** Performance of the model when the training data set is all normalized methods.

Classes	Proposed algorithm					DDAE			
	Accuracy	Precision	Recall	Specificity	F-score	Accuracy	Precision	Recall	F-score
Class 1	0.921	0.913	0.921	0.982	0.917	0.94	0.8	0.84	0.81
Class 2	0.950	0.947	0.950	0.9961	0.948	0.81	0.81	0.81	0.81
Class 3	0.906	0.965	0.906	0.998	0.934	0.88	0.85	0.82	0.83
Class 4	0.865	0.950	0.865	0.996	0.905	0.96	0.81	0.83	0.81
Class 5	0.983	0.9938	0.983	0.999	0.988	0.95	0.74	0.62	0.65
Class 6	0.989	0.965	0.989	0.998	0.977	0.93	0.83	0.8	0.81
Class 7	1.000	0.976	1.000	0.998	0.988	0.84	0.7	0.71	0.7
Class 8	0.978	0.959	0.978	0.994	0.968	0.84	0.7	0.61	0.62
Class 9	0.966	0.953	0.967	0.990	0.959				
Class 10	0.987	0.966	0.987	0.996	0.976				
Performance	0.954	0.959	0.954	0.995	0.956	0.894	0.780	0.755	0.755

## Discussion and conclusion

In this research, a robust algorithm is introduced to classify the audiograms based on the detected hearing levels since the steps to develop this algorithm is assessed at every single stage. It started with evaluating the unsupervised clustering using many evaluation criteria such as Calinski–Harabasz, Silhouette Coefficient and Davies–Bouldin criterion values for the selected number of clusters. Then, the authors justified their selection based on the nature of the audiograms, where, 10 clusters are chosen to capture the different shapes as possible. The aim of the authors’ first work was to classify audiograms according to different shapes taking into consideration how the filter bank is designed in hearing aids. This was followed by further improvement to the clusters by removing the wrongly assigned audiograms, which, led to higher values in different evaluation criteria^15^. The annotated data produced in the previous work is used as the training data set for the work in this paper. The authors normalized the data using 20 different normalization methods to increase the training data size in building a credible model, and then, selected 10 normalized data sets to train the model. This selection criterion emerged from the statistical analysis to remove any redundancy or any normalized data set that might lead to a change in the relative levels of each audiogram, which, will result in changes in audiograms’ shapes. This normalization process leads to a normalized data set of size 49 $\times$ 10 = 490 samples. Two other representations of the audiograms with negative and positive hearing levels are added to the normalized data sample, making it 588 samples. The authors have used Fine kNN as a classifier in MATLAB, where, k = 1 which makes the algorithm check all the samples in the nearest neighbors in the eigenspace one by one. Based on this assumption, it is believed that overfitting will not happen and different shapes will be detected accurately as there are fine detailed distinctions between the classes. However, Fine kNN has a high memory usage and a decreased model flexibility, which need to be considered in the real application where these criterias are required.

The supervised ML model proposed in this research is clearly outperforming the DDAE’s performance (based on recent research in this field) for both conditions: when the normalized data sets are trained individually, or, when trained with all the normalized data. The performance of the model; accuracy, precision, recall, specificity, and the F-score ($\beta$ = 1) are much better in both cases. The authors believe that the reason for the high accuracy of their classifier comes from the use of the nested features, where, it is studied thoroughly the limits of the basic features as indicated in Table 1. To the best of the authors’ knowledge, this is the first trial to classify audiograms according to the audiogram shape, which, will reduce the complexity of designing hearing aid filter bank and the process of configuring hearing aids. Configuring hearing aid using machine learning will reduce the number of responses and trials made by the audiologist and the patients in the conventional practice. It can be a great help for the children, the elderly, and individuals with dementia in reducing the stress of taking extensive tests. The used features to build the classifier are extracted based on hearing levels, and not based on the visual appearance of each audiograms. Visual appearance may lead to a wrong classification because it might differ due to the different practices in the clinics, where, different frequencies and threshold scales might be used. Based on the literature studies, features such as polynomial coefficients, residual, mapping functions, and nested features, are not used before by any other researchers. The usage of these features described the shape of audiograms precisely and led to a model with higher performance.

To conclude the findings in this study, the authors compared their previous work using spectral clustering in^15^ with the clusters produced by Gaussian Mixture Model (GMM) of^29^. Comparing these two unsupervised clustering classes, it can be deduced that C1, C2, C4, C5, C8, C9 and C10 are similar to N6, M10, N5, M6, M3, M9 and M8 respectively, but C3, C6 and C7 are not detected by the GMM clustering method for the NHANES and MEE databases. Parthasarathy’s clusters did not detect any clusters for severe hearing loss (C6) or severe to profound loss (C3). It is observed that the two classes C2 and C10 are for the steep configurations. The GMM method can cluster audiograms with the same mean and variance, and different shapes in the same cluster such as; 10, 20, 35, 35, 40, 45, 50, 70 and 10, 20, 25, 35, 50, 50, 55, 60, where, the first one is steep but the second is not. The difference in^15^ study is that the clustering method is selected using spectral clustering, to classify the audiograms according to the similarity in shape. Identifying the shape correctly is always a concern for the experts in the field. Consequently, the annotation currently given to the audiograms by the specialists in the field should be rechecked^30^ as the use of different unsupervised clustering methods results in different categories as shown in Fig. 1 and by the ones detected by the GMM of Parthasarathy^29^. The authors believe that the judgments based on clustering the audiograms using unsupervised ML, might lead to a change in the whole process of fixing the hearing loss, starting from the design of the hearing aid to configuring it according to the patient’s need.

This work can be used flexibly to classify the audiograms at any 8 frequencies between 125 Hz and 8 kHz. The audiograms in this study are to test the air conduction thresholds at eight test frequencies with or without masking in the non-test ear. Bone conduction thresholds are not considered in this study and can be considered in future studies. Another advantage of the proposed way is that the annotation process here is done using spectral clustering and not by the experts in the field, leading to a fixed criterion to classify audiograms. When the experts in the field are requested to annotate the data, their responses might differ as it depends on their perception, knowledge and their experience level which are very subjective and might be a source of confusion for the ML algorithm.

For future work, it is recommended to consider using another set of audiograms and data reduction analysis, different from the K-vector. Different hearing tests might be considered to produce accurate description of the patient hearing loss, and thus, provide suitable recommendations of action by the intelligent solutions. It can help the doctors to find the required information faster and draw a deep conclusion about the patient’s case. It will definitely save the time and lead the doctors to make a better decision. The diagnosis of the cause of hearing loss using different hearing tests can be a possible future application to be ventured on. Another application can be on studying the hearing levels at frequencies that are different from the current practices, and then, clustering the audiograms. It can lead to new classes, which can help in the diagnosis cases of tinnitus or noise damage. A common study can be conducted to build a generalized intelligent model based on the shapes that are found in our recent works and some of the other researchers’ work in the field. The authors forecast that there can be a change in currently used audiogram classes which will open a new perspective for hearing aid design.

## Data Availability

Approval to reuse the data from paper^14^ is obtained from SAGE Publishing at no cost for the life of the research work. The permission is obtained on September 2, 2021 via email for request RP-6079. SAGE Publishing allows the authors to use the data to publish any result based on the data given, but are not allowing the republishing of the data. The data of 60 audiograms are available in the paper, and can be used by contacting SAGE Publishing directly. The datasets used and/or analyzed during the current study are also available from the corresponding author on reasonable request.
